# Impact of Exercise Intensity on Systemic Oxidative Stress, Inflammatory Responses, and Sirtuin Levels in Healthy Male Volunteers

**DOI:** 10.3390/ijerph191811292

**Published:** 2022-09-08

**Authors:** Su-Youn Cho, Young-Soo Chung, Hyoung-Ki Yoon, Hee-Tae Roh

**Affiliations:** 1 Exercise Physiology Laboratory, Department of Physical Education, Yonsei University, Seoul 03722, Korea; 2 Department of Sports and Leisure Studies, School of Arts and Health, Myongji College, Seoul 03656, Korea; 3 School of Sports, College of Humanities, Soongsil University, Seoul 06978, Korea; 4 Department of Sports Science, College of Health Science, Sun Moon University, 70 Sunmoon-ro 221 beongil, Tangjeong-myeon, Asan-si 31460, Korea

**Keywords:** acute exercise, redox state, inflammation, sirtuin family

## Abstract

Exercise can induce anti-inflammatory and antioxidant effects, for which regulation of sirtuins (SIRTs) may be a major consideration for exercise prescription. The purpose of this study was to investigate the effects of acute aerobic exercise, in particular its intensity, on systemic oxidative stress, inflammatory responses, and SIRT levels. Twenty healthy, untrained males were recruited and randomly assigned to moderate-intensity (MI, 65% VO_2_max, *n* = 10) and high-intensity (HI, 85% VO_2_max, *n* = 10) exercise. Blood samples were obtained pre-, immediately post-, and 1 h post-exercise for measurements of malonaldehyde (MDA), superoxide dis-mutase (SOD), interleukin (IL)-1β, IL-6, tumor necrosis factor (TNF)-α, SIRT-1, SIRT-2, and SIRT-3. Overall, MDA, SOD, IL-6, SIRT-1, and SIRT-3 levels were significantly increased at post-exercise compared with pre-exercise regardless of exercise intensity (*p* < 0.05). The HI group had significantly higher MDA, SOD, and IL-6 levels than the MI group at post-exercise (*p* < 0.05), whereas no significant differences were observed in the IL-1β, TNF-α, and SIRT-2 levels (*p* > 0.05). Altogether, these findings suggest that exercise-induced oxidative stress and inflammatory responses may be dependent on exercise intensity. Moreover, activation of inflammatory cytokines and SIRT family members may be dependent on the intensity of the exercise.

## 1. Introduction

Exercise, including regular physical activity, has been proven to be effective in preventing and ameliorating the symptoms of various metabolic syndromes and cardiovascular diseases, such as obesity, type 2 diabetes, and hyperlipidemia [1,2]. High oxidative stress (OS) and chronic low-grade systemic inflammation in the body are involved in the pathogenesis of various diseases, and exercise was shown to regulate these events. For example, regular physical training was reported to reduce OS levels by promoting the antioxidant capacity and has anti-inflammatory effects by reducing the levels of several pro-inflammatory cytokines [3,4]. Roh and So [5] demonstrated that aerobic exercise for 8 weeks effectively promotes the activity of the antioxidant enzyme superoxide dismutase (SOD), whereas it significantly decreases the levels of reactive oxygen species (ROS) in obese men. Roh et al. [6] also reported that the serum inflammatory markers of obese elderly people can be significantly reduced after 12 weeks of resistance training. Furthermore, regular exercise was reported as a valuable strategy to tackle aging-related diseases, such as neurodegenerative diseases, diabetes, and cardiovascular diseases, by regulating sirtuins (SIRTs) involved in mitochondrial energy homeostasis, antioxidant activity, proliferation, and DNA repair [7,8,9]. However, most of these previous studies [7,10,11] only confirmed the changes in SIRTs by mediating exercise in animal models. Steiner et al. [10] reported a significant increase in SIRT-1 mRNA in the soleus muscle and various regions of the brain (cortex, frontal lobe, hippocampus, hypothalamus, among others) in 8-week-old male mice after performing endurance exercise in for 8 weeks. Moreover, Hokari et al. [11] reported that SIRT-3 levels increased significantly in the soleus and plantaris muscles in male rats after 3 weeks of treadmill running.

It has been demonstrated that acute exercise can induce the generation of ROS by increasing the metabolic rate in mitochondria and eventually increasing the OS level [12]. It has been suggested that exercise-induced OS may be dependent on exercise intensity [12]. Strenuous exercise not only induces OS, but also can elicit tissue damage, including to skeletal muscle, which can cause an inflammatory response [12,13]. Thiobarbituric acid reactive substances, 8-oxo-2′-deoxyguanosine, malondialdehyde (MDA), and 4-hydroxynonenal (4-HNE), which reflect lipid peroxidation, have been reported as blood biomarkers of OS [12,14]. Pro-inflammatory cytokines, such as interleukin (IL)-1β, IL-6, IL-10, and tumor necrosis factor (TNF)-α, have been suggested as markers reflecting the inflammatory response [15]. However, different cytokines may respond differently to exercise [15], and consistent results are not available. In addition, it has been reported that increased phosphorylation of AMP-activated protein kinase (AMPK) may play a major role in the activity of SIRT-1 [16], and that SIRT-3 is associated with antioxidant capacity [17]. Although this suggests the possibility that different exercise intensities may affect the activity of SIRTs, the related studies are limited.

Intensity, along with type, frequency, and time (duration), are the main features of exercise that should be considered to maximize the subclinical effect of exercise therapy. Nonetheless, to date, no changes in SIRTs have been reported in humans while considering the levels of acute exercise intensity. Therefore, this study aimed to investigate the changes in oxidative stress, inflammatory response, and SIRTs according to differences in exercise intensity by facilitating treadmill running of different intensity in adult males.

## 2. Methods

### 2.1. Participants

Twenty healthy males who did not perform any regular exercise program, had no injuries nor diseases, and were non-smokers, as determined by a health screening questionnaire, were enrolled in the study. The participants were randomly assigned to the moderate-intensity (MI) or high-intensity (HI) groups (*n* = 10 each), in which treadmill running was performed at an exercise intensity of 65% and 85% maximal oxygen uptake (VO_2_max), respectively. All participants provided written informed consent prior to participating in the study. The ethics committee of the Dong-A University approved the study protocol (approval No. 2-104709-AB-N-01-201710-HR-046-02). The physical and clinical characteristics of the participants are provided in Table 1.

### 2.2. Preliminary Tests

Preliminary tests were performed 1 week before the exercise trials. Height and body composition were measured with a multifrequency bioimpedance analysis device (Accuniq BC720; SELVAS Healthcare, Daejeon, Korea). Resting blood pressure, heart rate, and SpO_2_ were measured with a multiparameter patient monitor (M30; Mediana, Kangwon, Korea). VO_2_max was measured with a respiratory gas analyzer (Quark CPET; Cosmed, Rome, Italy) by breath-by-breath technique according to the Bruce treadmill protocol [18].

### 2.3. Treadmill Running Test Protocol

Based on the measured VO_2_max values, treadmill running tests were performed with two exercise intensity groups: 65% and 85% VO_2_max, as previously described by Roh et al. [19]. Specifically, treadmill running conditions were adjusted based on the oxygen consumption (VO_2_). If a subject’s VO_2_ reached the corresponding target exercise intensity, the grade and speed of the treadmill were adjusted to maintain VO_2_ in the steady state. When the total energy consumption indicated by the respiratory gas analyzer reached 300 kcal, treadmill running was terminated.

### 2.4. Blood Collection and Analyses

Intravenous blood samples were obtained pre-, immediately post-, and 1 h post-exercise in vacutainer tubes treated with serum separation and ethylenediamine tetra-acetic acid tubes (Becton Dickinson, Franklin Lakes, NJ, USA) for serum and plasma isolation, respectively. All collected serum and plasma were preserved at −80 °C until analysis. The malonaldehyde (MDA) levels and SOD activity in the plasma were determined by the colorimetric assays as previously described by [20], using commercially available BIOXYTECH LPO-586 (#21012; Oxis International, Portland, OR, USA) and Superoxide Dismutase Assay (#CM706002; IBL International, Hamburg, Germany) kits according to the manufacturer’s protocol. The analyses of IL-1β, IL-6, TNF-α, SIRT-1, SIRT-2, and SIRT-3 levels in the sera were determined by the enzyme-linked immunosorbent assay (ELISA). IL-1β (#HSLB00D), IL-6 (#HS600C), and TNF-α (#HSTA00E) ELISA kits were purchased from R&D Systems (Minneapolis, MN, USA). SIRT-1 (#CSB-E15058h) and SIRT-3 (#CSB-EL021341HU) ELISA kits were purchased from CUSABIO (Wuhan, China). SIRT-2 ELISA kit (#MBS2530369) was purchased from Mybiosource (San Diego, CA, USA). The results were read at 450 nm using a microplate reader (Tecan Sunrise; TECAN GmbH, Salzburg, Austria).

### 2.5. Statistical Analyses

All statistical analyses were performed using the SPSS Statistics version 26.0 (IBM Corp., Armonk, NY, USA), and all data are given as means ± standard deviation (SD). Tests of normality for all measured values were performed using the one-sample Kolmogorov–Smirnov test. Two-way repeated measures analysis of variance (ANOVA) was performed to examine the interaction effects of time (pre-, immediately post-, and 1 h post-exercise) and experimental groups (MI and HI) on the measured variables. Simple main effects were assessed using one-way ANOVA. Levels of significance were considered at *p* < 0.05.

## 3. Results

### 3.1. Changes in Oxidative Stress (OS) Markers

**Figure 1:**
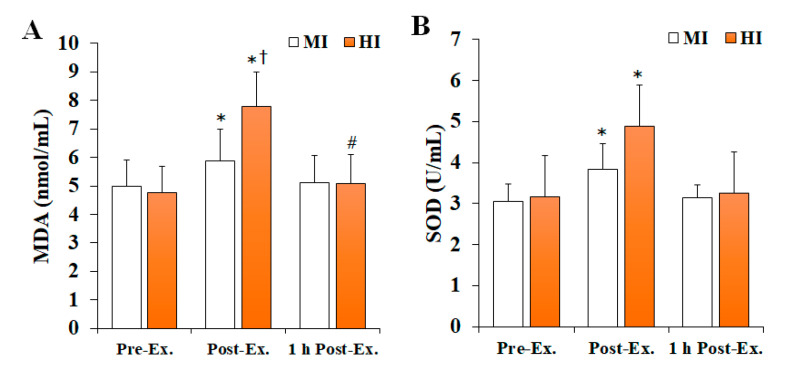
The changes in OS markers after treadmill running at different exercise intensities. Values are presented as mean ± SD. (**A**) MDA: malonaldehyde, (**B**) SOD: superoxide dismutase, MI: moderate-intensity, HI: high-intensity, Ex: exercise, * significant difference with pre-exercise and 1 h post-exercise within group (*p* < 0.05), ^#^ significant difference with pre-exercise within group (*p* < 0.05), ^†^ significant difference with MI group within time (*p* < 0.05).

Changes in OS markers after treadmill running at different exercise intensities are shown in Figure 1. Noteworthily, MDA levels (*F* = 14.723, *p* < 0.001, *ηp*^2^ = 0.450) and SOD activity (*F* = 8.342, *p* = 0.001, *ηp*^2^ = 0.317) significantly changed according to the time and intensity of the exercise. Specifically, both MDA levels and SOD activity were significantly increased at post-exercise compared with pre-exercise (*p* < 0.05), and then significantly decreased at 1 h post-exercise (*p* < 0.05), regardless of the exercise intensity. Nevertheless, the HI group had significantly higher MDA levels at 1 h post-exercise than pre-exercise (*p* < 0.05), and significantly higher MDA levels than the MI group at post-exercise (*p* < 0.05). Furthermore, the HI group had significantly higher SOD activity than the MI group at post-exercise (*p* < 0.05).

### 3.2. Changes in Inflammatory Markers

**Figure 2:**
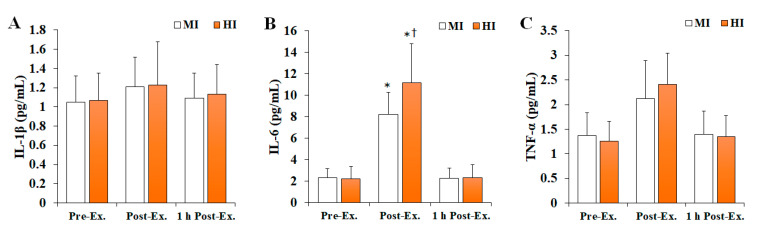
The changes in inflammatory markers after treadmill running at different exercise intensities. Values are presented as mean ± SD. (**A**) IL-1β: interleukin-1β, (**B**) IL-6: interleukin-6, (**C**) TNF-α: tumor necrosis factor-α, MI: moderate-intensity, HI: high-intensity, Ex: exercise, *: significant difference with pre-exercise and 1 h post-exercise within group (*p* < 0.05), †: significant difference with MI group within time (*p* < 0.05).

Changes in inflammatory markers after treadmill running at different exercise intensities are shown in Figure 2. Overall, IL-6 levels (*F* = 6.335, *p* = 0.004, *ηp*^2^ = 0.206) were found to significantly change depending on the time and intensity of the exercise, being significantly increased at post-exercise compared with pre-exercise (*p* < 0.05), and then significantly decreased at 1 h post-exercise (*p* < 0.05) in both MI and HI groups. In addition, the HI group had significantly higher IL-6 levels than the MI group at post-exercise (*p* < 0.05). In contrast, no significant differences were observed regarding IL-1β (*F* = 0.008, *p* = 0.992, *ηp*^2^ = 0.001) and TNF-α (*F* = 1.650, *p* = 0.206, *ηp*^2^ = 0.084) levels.

### 3.3. Changes in SIRT Levels

**Figure 3:**
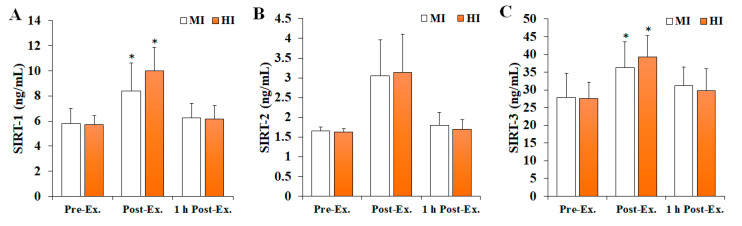
The changes in SIRT levels after treadmill running at different exercise intensities. Values are presented as mean ± SD. (**A**) SIRT-1: sirtuin 1, (**B**) SIRT-2: sirtuin 2, (**C**) SIRT-3: sirtuin 3, MI: moderate-intensity, HI: high-intensity, Ex: exercise, * significant difference with pre-exercise and 1 h post-exercise within group (*p* < 0.05).

Changes in SIRT levels after treadmill running at different exercise intensities are shown in Figure 3. The levels of SIRT-1 (*F* = 4.535, *p* = 0.018, *ηp*^2^ = 0.201) and SIRT-3 (*F* = 3.522, *p* = 0.040, *ηp*^2^ = 0.164) were found to significantly change according to the time and intensity of the exercise. Specifically, they were significantly increased at post-exercise compared with pre-exercise (*p* < 0.05), and then significantly decreased at 1 h post-exercise (*p* < 0.05) in both MI and HI groups. In contrast, no significant differences were observed in SIRT-2 levels (*F* = 0.125, *p* = 0.883, *ηp*^2^ = 0.007).

## 4. Discussion

Regular physical activity, especially aerobic training, can alleviate OS levels in the body by inducing an increase in antioxidant enzyme activity [21]. In contrast, increased oxygen demands by active muscles due to acute exercise leads to increased oxygen consumption and mitochondrial activity, which consequently induces OS by increasing the production of reactive oxygen species, such as the superoxide radical (O_2_˙^−^) [12,22]. Lipids have been proposed as the most relevant biomolecule among the various biological targets of OS [23]. Both 4-HNE and MDA reflect lipid peroxidation and have been reported as one of the blood indicators of exercise-induced OS [14,24,25]. In this study, plasma MDA levels were analyzed to confirm the changes in OS according to exercise intensity. The results of the present study showed that plasma MDA levels significantly increase in response to exercise regardless to its intensity (MI or HI), which agrees with previous studies [24,26] that reported a significant increase in plasma MDA levels after acute exercise. This phenomenon is believed to result from free radicals produced by exercise that will in turn promote lipid peroxidation. Souissi et al. [26] reported that plasma MDA levels increase significantly after intermittent running exercise at 75% of maximal aerobic speed. Moreover, Spirlandeli et al. [24] showed that plasma MDA levels were significantly increased in eight healthy and well-trained males upon acute exercise intervention, as determined by three different methods (high-performance liquid chromatography, thiobarbituric acid reactive species, and 1-methyl-2-phenylindole analyses), which suggested that MDA could be a valuable biomarker of lipid peroxidation following acute exercise. In this study, the MDA levels were also found to be significantly higher in the HI group compared with the MI group at post-exercise, and at 1 h post-exercise compared to pre-exercise. These findings suggest that plasma MDA levels increased by acute exercise may be dependent on exercise intensity and supports the findings of previous studies, which reported a significant increase in plasma MDA levels after relatively high-intensity acute exercise [27,28]. Indeed, Sureda et al. [27] reported that lymphocyte MDA concentrations increased significantly only after high-intensity exercise in 18 male soccer players, and Seifi-Skishahr et al. [28] showed that serum MDA levels significantly increased at 2 h post-exercise for high-intensity (75% VO_2_max), whereas no changes were noticeable between pre-exercise and 2 h post-exercise with moderate-intensity (60% VO_2_max) exercise.

The body has a complex antioxidant defense system that includes SOD, which reduces superoxide radicals to hydrogen peroxide (H_2_O_2_) and water (H_2_O), as well as catalase and glutathione peroxidase [29,30]. Herein, plasma SOD activity was evaluated to assess the antioxidant enzyme status according to exercise intensity. Overall, SOD activity was found to be significantly increased in both MI and HI groups at post-exercise compared with pre-exercise. These results agree with those of the previous studies, which reported a significant increase in plasma SOD activity after acute exercise, suggesting that SOD activity may increase during exercise at moderate- or high-intensity. Thirupathi et al. [31] suggested that exercise can induce an increase in the activity of antioxidant enzymes, such as SOD, with concomitant production of ROS. Moreover, Berzosa et al. [32] showed that plasma SOD activity increases significantly after submaximal and maximal exercise in healthy adult males. In the study by Shin et al. [33], the participants underwent treadmill running until 400 kcal were consumed while 60% and 80% VO_2_max were achieved. Interestingly, in these conditions, the plasma MDA levels were found to increase significantly only after 80% VO_2_max exercise, whereas SOD activity increased significantly regardless of the intensity of the exercise. In addition, it is thought that the increase in SOD in response to exercise may be related to activation of nuclear factor erythroid 2-related factor 2 (Nrf2)/antioxidant response element (ARE) signaling. This redox-sensitive signaling system plays an important role in the maintenance of cellular homeostasis under stress and inflammatory conditions [34]. Muthusamy et al. [35] reported that acute exercise stress activated Nrf2/ARE signaling in an animal model, while Done et al. [36] demonstrated that acute exercise in humans can increase Nrf2 protein levels in peripheral blood mononuclear cells.

Acute exercise can induce oxidative stress but also transient inflammatory response, as denoted by changes in the levels of several pro-inflammatory cytokines, including IL-1β, IL-6, IL-10, and TNF-α [37,38]. Herein, serum IL-1β, IL-6, and TNF-α levels were analyzed to verify the inflammatory response according to exercise intensity. Noteworthy, IL-6 levels were found to be significantly increased significantly in both the MI and HI groups at post-exercise compared with pre-exercise, with the increase at post-exercise in the HI group being greater than in the MI group, which agrees with previous findings in acute exercise settings that suggest that exercise-induced inflammatory response, in addition to OS, is dependent on exercise [39,40,41]. Ribeiro-Samora et al. [40] suggested that exercise intensity is an important factor for controlling the degree of exercise-induced OS and inflammatory response. Furthermore, Fischer [41] reported that the IL-6 response according to exercise is dependent on its intensity and that increased ROS formation can activate transcription factors that regulate IL-6 synthesis. However, no significant difference in IL-1β and TNF-α levels were observed in the present study, which may be explained by differences in the sensitivity of pro-inflammatory cytokines towards exercise. According to Kasapis and Thompson [42], IL-6 levels can be increased by up to 100-fold after strenuous exercise, which the most prominent and faster cytokine response to exercise detected, whereas changes in TNF-α are rarely observed.

SIRTs (SIRT-1–7) are NAD^+^-dependent deacetylases or ADP-ribosyl-transferases that can act as regulators of OS, DNA repair, transcription, cell cycle progression, and metabolism [9]. Regular exercise has been reported to positively affect the activity and/or expression of SIRTs and may be involved in the improvement of biogenesis, mitochondrial function, and maintenance of the antioxidant system [8]. However, studies verifying changes in serum SIRT levels due to acute exercise are limited. Therefore, this study analyzed serum SIRT-1, SIRT-2, and SIRT-3 levels to verify changes in SIRTs due to acute exercise and differences in exercise intensity. Overall, SIRT-1 and SIRT-3 were found to significantly increase in both the MI and HI groups at post-exercise compared with pre-exercise, whereas SIRT-2 levels remain unchanged. These results agree with previous reports [8,43] that described a significant increase in SIRT-1 after acute exercise and that changes in SIRTs due to exercise are limited to SIRT-1 and SIRT-3. Vargas-Ortiz et al. [8] reported that SIRT-1 and SIRT-3 can be altered by exercise, with SIRT-1 being activated after a single load of exercise and increased AMPK phosphorylation playing a major role in this mechanism. Furthermore, SIRT-3 deacetylates forkhead box O3 (FOXO3) and is involved in cell differentiation and survival. In particular, activation of FOXO3 by SIRT-3 promotes the reduction in intracellular ROS via the activity of the antioxidant enzymes SOD2 and catalase [44,45]. Considering that the herein described significantly increased SIRT-3 levels immediately after exercise followed the same trend as that of SOD, it is reasonable to speculate that the increase in SIRT-3 levels results from an effort of the body to neutralize exercise-induced OS.

The strength of this study is considered to be the comprehensive verification of changes in the oxidant–antioxidant balance, inflammatory response, and SIRTs in humans due to the difference between acute exercise and exercise intensity. However, this study has some limitations. First, the participants in this study were limited to healthy adult males, so the results could not be generalized to the entire human population. Second, changes in variables according to exercise intensity were not verified at the cellular level. Finally, we could not control the intake of nutrients which could affect the variables.

## 5. Conclusions

In conclusion, this study provides further evidences that acute exercise can induce transient oxidative stress and inflammatory response in an exercise intensity-dependent manner. In addition, SIRT-1 and SIRT-3 levels can be increased by acute exercise regardless of its intensity, whereas SIRT-2 remains unchanged, suggesting that there may be differences in the activation of SIRT family members with acute exercise.

## Figures and Tables

**Table 1:** The physical and clinical characteristics of the participants at baseline.

Variables	MI (*n* = 10)	HI (*n* = 10)	*p* ^#^
Age (years)	21.60 ± 1.43	20.70 ± 1.34	0.163
Height (cm)	175.56 ± 3.57	178.78 ± 4.70	0.102
Weight (kg)	73.34 ± 6.05	75.36 ± 7.80	0.526
BMI (kg/m^2^)	23.83 ± 2.25	23.60 ± 2.44	0.822
PBF (%)	21.67 ± 6.65	20.21 ± 4.50	0.572
SBP (mmHg)	120.70 ± 9.09	124.60 ± 5.93	0.271
DBP (mmHg)	76.30 ± 7.83	80.70 ± 5.40	0.161
HRrest (beats/min)	73.70 ± 10.70	70.70 ± 5.77	0.445
SpO_2_ (%)	98.40 ± 0.70	98.80 ± 0.63	0.196
VO_2_max (mL/kg/min)	48.48 ± 2.88	48.94 ± 3.09	0.732
MDA (nmol/mL)	4.99 ± 0.93	4.77 ± 0.90	0.608
SOD (U/mL)	3.06 ± 0.42	3.17 ± 0.43	0.544
IL-1β (pg/mL)	1.05 ± 0.27	1.07 ± 0.28	0.842
IL-6 (pg/mL)	2.31 ± 0.83	2.23 ± 1.12	0.861
TNF-α (pg/mL)	1.37 ± 0.46	1.26 ± 0.40	0.563
SIRT-1 (ng/mL)	5.79 ± 1.20	5.70 ± 0.73	0.844
SIRT-2 (ng/mL)	1.66 ± 0.10	1.63 ± 0.08	0.458
SIRT-3 (ng/mL)	27.86 ± 6.77	27.52 ± 4.57	0.899

Values are presented as mean ± SD. BMI: body mass index, PBF: percentage of body fat, SBP: systolic blood pressure, DBP: diastolic blood pressure, HRrest: resting heart rate, SpO_2_: transcutaneous arterial oxygen saturation, VO_2_max: maximal oxygen uptake, MDA: malonaldehyde, SOD: superoxide dismutase, IL-1β: interleukin-1β, IL-6: interleukin-6, TNF-α: tumor necrosis factor-α, SIRT-1: sirtuin 1, SIRT-2: sirtuin 2, SIRT-3: sirtuin 3, MI: moderate-intensity, HI: high-intensity. ^#^ Determined using the independent *t*-test.

## Data Availability

Data generated and analyzed during this study are included in this article. Additional data are available from the corresponding author on request.
